# Cytokines in the Respiratory Airway as Biomarkers of Severity and Prognosis for Respiratory Syncytial Virus Infection: An Update

**DOI:** 10.3389/fimmu.2019.01154

**Published:** 2019-06-04

**Authors:** Yaneisi Vázquez, Liliana González, Loreani Noguera, Pablo A. González, Claudia A. Riedel, Pablo Bertrand, Susan M. Bueno

**Affiliations:** ^1^Millennium Institute on Immunology and Immunotherapy, Departamento de Genética Molecular y Microbiología, Facultad de Ciencias Biológicas, Pontificia Universidad Católica de Chile, Santiago, Chile; ^2^Millennium Institute on Immunology and Immunotherapy, Departamento de Ciencias Biológicas, Facultad de Ciencias de la Vida, Universidad Andrés Bello, Santiago, Chile; ^3^División de Pediatría, Unidad de Enfermedades Respiratorias Pediátricas, Facultad de Medicina, Pontificia Universidad Católica de Chile, Santiago, Chile

**Keywords:** biomarker, cytokines, LRTI, hRSV, severity, prognosis

## Abstract

The human respiratory syncytial virus (hRSV) is one of the most important causes of upper and lower respiratory tract infections in children and the main cause of bronchiolitis worldwide. Disease manifestations caused by hRSV may vary from mild to severe, occasionally requiring admission and hospitalization in intensive care units. Despite the high morbidity rates associated to bronchiolitis, treatment options against hRSV are limited and there are no current vaccination strategies to prevent infection. Importantly, the early identification of high-risk patients can help improve disease management and prevent complications associated with hRSV infection. Recently, the characterization of pro- and anti-inflammatory cytokine patterns produced during hRSV-related inflammatory processes has allowed the identification of potential prognosis biomarkers. A suitable biomarker should allow predicting the severity of the infection in a simple and opportune manner and should ideally be obtained from non-invasive samples. Among the cytokines associated with hRSV disease severity, IL-8, interferon-alpha (IFN-alpha), and IL-6, as well as the Th2-type cytokines thymic stromal lymphopoietin (TSLP), IL-3, and IL-33 have been highlighted as molecules with prognostic value in hRSV infections. In this review, we discuss current studies that describe molecules produced by patients during hRSV infection and their potential as biomarkers to anticipate the severity of the disease caused by this virus.

## Introduction

The human respiratory syncytial virus (hRSV) is a viral agent predominantly involved in acute lower respiratory tract infections (LRTIs), frequently associated to bronchiolitis and pneumonia in children and infants (1, 2). HRSV is responsible for approimately 60% of all LRTIs in children under 5 years old and causes more than 80% of the reported cases in infants (3, 4). At the age of 2 years, almost all children have been infected with hRSV at least once, and disease severity among these children may vary from mild to severe manifestations, sometimes requiring hospitalization with oxygen administration or admission into intensive care units (5, 6). Moreover, hRSV infection may cause exacerbated airway diseases and has been associated with recurrent wheezing and asthma development (7, 8).

Several attempts to reduce the impact of hRSV-LRTI in health-care have been made. The first vaccine trial for hRSV was based on a formalin-inactivated hRSV formulation (FI-hRSV) in the 1960's, but this formulation was unable to generate an effective immune response and conversely produced an exacerbated disease in children after hRSV infection (9). Since this first failed attempt, several other vaccination strategies have been addressed, ranging from live-attenuated viral approaches to recombinant proteins, as well as recombinant organisms using both, viral and bacterial vectors as immunoadjuvants (10). It is important to highlight a growing number of clinical vaccine trials in the last decades aiming to identify a protective approach (phase I and II, ClinicalTrials.gov 2017: Identifier: NCT03213405 and 2018 Identifier: NCT03636906) (2, 11). However, despite the significant progress achieved in this field, until now there are no commercially available vaccines against hRSV (12).

Regarding hRSV disease management in high-risk groups, prophylaxis based on neutralizing monoclonal antibodies has been implemented to prevent severe manifestations associated to hRSV-LRTI (13–15). Palivizumab and Motavizumab are two humanized monoclonal antibodies generated against the hRSV fusion protein F that have shown efficacy in preventing hRSV infection and the capacity to decrease the rate of hospitalization of hRSV-infected infants (16). However, only Palivizumab has been licensed to be used as a therapy against hRSV severe infections associated with bronchiolitis and pneumonia. Yet, it is unable to induce long-lasting protection in those treated and the costs associated to its use make difficult the implementation of this strategy as a first treatment option (14). Despite the existence of the neutralizing antibodies described above as prophylactic and therapeutic strategies, these approaches do not work as vaccines. Hence, to date there is no successful and affordable strategy available to control hRSV outbreaks, which represent an important public health problem worldwide (17, 18).

Therefore, strategies to prevent complications derived from hRSV infection and improve disease management are needed. Based on this premise, early diagnosis, and prediction of disease severity has raised considerable interest in researchers and the search for biological biomarkers to predict disease severity during hRSV infection. In this review we discuss the latest studies available in PubMed on potential prognosis biomarkers and revise the feasibility of including them during routine hRSV diagnosis.

## Characteristics and Pathogenesis of HRSV

**Figure 1:**
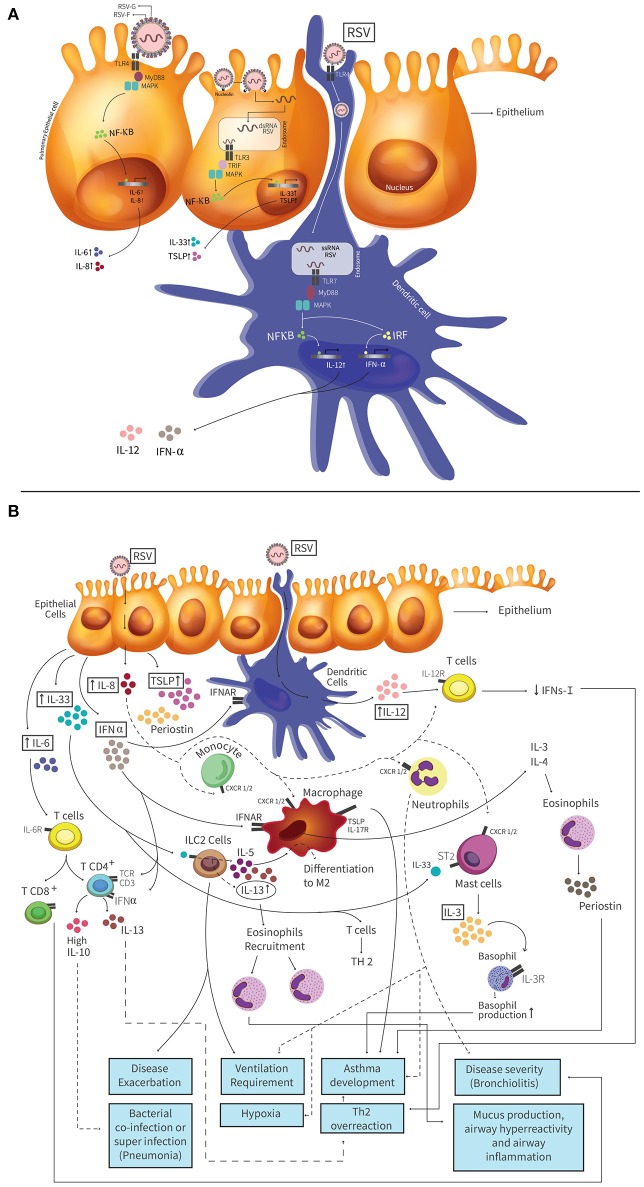
Pathogenesis of hRSV and molecules with a biomarker potential induced in the airways during hRSV infection. **(A)** HRSV attaches to airway epithelial cells and this binding is mediated by the interaction between the fusion (F) or glycoprotein (G) protein of hRSV. Toll-like receptor 4 **(TLR4)** is expressed on AECs and it is involved in the hRSV entry. When hRSV F protein binds to TLR4, this triggers a cascade of signaling, where the protein myeloid differentiation primary response 88 (MyD88) is activated. The activation of MyD88 leads to activation of mitogen-activated protein kinase (MAPK), and the NF-kB transcription factor. Activated NF-kB translocates to the nucleus and promotes the production of Th1 cytokines (like as TNF-α, IL-6, and IL-8). **Nucleolin** is a protein located on the cell surface that is also involved in the entry process of hRSV, which generates a fusion between host cell membrane and the virus. This fusion allows the entry of the viral genetic material to the cell, and the binding of dsRNA to TLR3. TLR3 triggers a cascade of signaling by the TIR-domain-containing adapter-inducing interferon-β (TRIF), MAPKs and NF-kB transcription factor. This signaling pathway promotes the IL-33 and TSLP production. HRSV also can infect Dendritic Cells **(DCs)** and the virus mediates its entry by TLR4 receptor, present on the surface of the DC. DCs are then infected and the genetic material of the virus enters the cell. dsRNA binds TLR7 receptor, present in the endosome produced by the fusion, which one TLR3 triggers a cascade of signaling by the MyD88 protein, MAPKs and NF-kB transcription factor or interferon-regulatory factor (IRF). Those signaling pathways promote the IL-12 and IFN-α production, respectively. **(B)** Infected AECs secrete several cytokines and chemokines that have been described as potential biomarkers. High **IL-33** levels are produced by AECs and cells expressing ST2 receptor, such as ILC2s, respond to IL-33 through the production of IL-5 and IL-13, which promote the recruitment of eosinophils that generate disease exacerbation and is associated to ventilation requirement. The mast cells also express the ST2 receptor and when IL-33 binds to these receptors the production of IL-3 is promoted. AECs produce high levels of **IL-8**, promoting the recruitment neutrophils to the infection site, that could generate a degree of hypoxia, ventilation requirement and asthma development. **TSLP** production is mediate by AECs. This cytokine is recognized by the receptor TSLPR, which is expressed by macrophages, generating an exacerbation of the disease and asthma. **Periostin** is produced by AECs or eosinophils. This protein increases the expression of inflammatory mediators. Deposits of periostin in the lung is associated with increased severity of asthma. **IL-6** is produced by AECs and promotes a Th2 response. This cytokine is involved in the promotion of naïve differentiation to CD4^+^ and CD8^+^ T cell. CD4^+^ T cells trigger the IL-13 production and Th2 overreaction response. CD8^+^ T cells increase the disease severity. **IFN-α** is produced by pDCs and AECs. At late times of infection, high levels of this cytokine produce high IL-10 levels by T cells. **IL-12** is produced by pDCs and promotes the differentiation of naive T cells into Th1 cells and induces weak IFN-γ-production by T cells. This low IFN-γ-production generate a Th2 overreaction response. **IL-3** promotes basophil and eosinophil production, triggering inflammatory and allergic diseases as asthma. **IL-13** is produced by ILC2 cells and CD4^+^ T cells, among other. High IL-13 levels result in a Th2 overreaction response and the recruitment of eosinophils that generate exacerbated mucus production, airway hyperreactivity and inflammation. Different lines (dotted and solid) were used to facilitate understanding of the figure and the different signaling pathways involved.

HRSV is an enveloped, negative, single-stranded RNA virus belonging to the *Pneumoviridae* family (19, 20). The genome of hRSV has 10 genes encoding 11 proteins required for the replicative cycle of hRSV in infected cells (21, 22), as well as for the modulation of the host immune response (23). Two hRSV subtypes have been identified, A and B, with the subtype A mostly associated to outbreaks during winter in countries with temperate climates (24, 25). hRSV is transmitted by direct contact or aerosol particles and once in the airways it replicates in mucosal epithelial cells, starting in the upper respiratory tract and then continuing to the lower respiratory tract (26). When hRSV arrives to the lower respiratory tract, viral antigen recognition by innate immune cells induce an inflammatory response, a process that is the result of complex interactions between the pathogen and host factors (27, 28). Lung inflammation is likely the result of a non-effective activation of the innate immunity by hRSV infection, mainly leading to Th2 and/or Th17 immune responses that generate mucus overproduction in the airways and enhance the inflammatory immune response in this tissue, leading to lung immunopathology (29, 30). After airway epithelial cells (AECs) recognize hRSV components (e.g. F protein and virus-related nucleic acids) through Toll-like receptors (i.e., TLR3 or 4) (Figure 1A) and retinoic-acid inducible gene I (RIG-I) receptors, signaling pathways activate transcription factors, such as interferon-regulatory factor 3 (IRF-3), and nuclear factor κB (NF-κB) (Figure 1A). In turn, these proteins promote the transcription of several anti-viral genes and soluble molecules (30, 31). In response to hRSV infection, AECs produce proinflammatory molecules such as type-I and type-III interferons (IFN) (31, 32). IFNs bind to IFN receptors (e.g., IFNAR) located on the surface of target cells and activate signaling pathways via Signal Transducer and Activator of Transcription 1 (STAT-1) and STAT-2 transcription factors. Ideally, STAT will bind to IFN-regulatory factors for a complete promotion in the transcription of interferon-stimulated genes (ISGs). Concomitantly, pro-inflammatory cytokines such as IL-6, tumor necrosis factor alpha (TNF-α) and chemokines (e.g., CXCL8, CCL3, CCL2, and CCL5) are induced and secreted to the extracellular medium. Importantly, some of these molecules (i.e., CCL2 and CCL5) will promote the recruitment of leukocytes (i.e., monocytes and neutrophils), dendritic cells, macrophages, natural killer cells, and CD4^+^ T cells to the site of infection (31, 32).

Effective clearance of the hRSV requires a balanced Th1 and Th2 adaptive immune response, which promotes IFN-γ production by cytotoxic CD8^+^ T cells (27, 33). However, during hRSV infection a weak type-I IFN response is elicited in the host, whereby viral replication is effective in infected cells and a pro-inflammatory Th2-response is generated (34) (Figures 1A,B). Because hRSV infection does not produce an effective memory response that confers protective immunity to subsequent viral exposure, re-infections are very frequent which lead to hyperreactivity, recurrent wheezing and an increased susceptibility of developing asthma (35).

## Clinical Manifestations of HRSV Infection

Clinical manifestations of LRTI caused by hRSV might vary depending on the individual's co-morbidity, age or sex, air pollution exposure, parental asthma history or previous infections, among others (2, 36). HRSV-LRTI might be accompanied by nasal congestion, rhinorrhea, cough, wheezing and shortness of breath (36, 37), with an increased risk of subsequent wheezing episodes that can last for several years after acute infection. Indeed, pathology induced in the airways by respiratory viruses is characterized by alterations in the respiratory epithelium, which stimulates the production of pro-inflammatory cytokines and chemokines that promote the infiltration of immune cells into the lungs (38, 39). In some cases, this response might become exacerbated and bring temporary or lifetime changes in the lungs, leading to the recurrent wheezing episodes and asthma (3, 40). Although most viral infections induce a transient airway hyperresponsiveness (41, 42), those with a history of atopy or asthma might display enhanced virus-related inflammation with significant airway obstruction leading to a more severe disease (43, 44). Therefore, the identification of hRSV-infected patients susceptible to develop more severe diseases would be important for performing better clinical decisions.

## Diagnosis of HRSV Infection

Early clinical diagnosis of hRSV infection could help to improve the care management of patients with respiratory infections and anticipate severe outcomes, according to the clinical predisposing factors, such as age. Currently, the available methods for hRSV diagnosis include tests that are based on molecular, virologic, or immunologic diagnostic.

Nowadays, the most used methods for hRSV diagnosis are based on direct immunofluorescence (DIF), reverse transcriptase-PCR (RT-PCR), immunochromatographic assay (CIA) and enzyme immune-assay (EIA). Other more complex methodologies that have been used more frequently in the last years are based in the detection of multiple analytes in high-complexity multiplex assays (such as Luminex or Affimetrix), as these approaches are faster than viral culture (11, 45, 46). Some molecular assays, such as RT-PCR and Luminex have high diagnostic sensitivity as compared to cell culture technique, but only RT-PCR is used as reference technique (47). Although RT-PCR is the fastest, its implementation is expensive as compared to DIF, EIA, or CIA assays. However, while the latter are low cost and fast, their sensitivity is lower than that of RT-PCR or Luminex and, in some cases (i.e., DIF), the interpretation of the results is somewhat subjective and requires technical skills, time, and expertise (47).

Immunologic diagnosis of hRSV is based on the characterization of cellular and cytokine/chemokine profiles (48, 49). In this case, flow cytometry is the main technique used to identify the cell types present in the bronchoalveolar lavage fluid (BALF) and peripheric blood samples of patients with hRSV infection. Cells recruited to the lungs include neutrophils, dendritic cells, T and B cells, alveolar macrophages and monocytes (10, 35). Clinical studies with hRSV infected children have shown an increased amount of neutrophils (CD11b^+^, CD18^+^, and CD54^+^) (50), alveolar macrophages (expressing TNF-α) (51), monocytes (CD69^+^) (52) and B cells (53) infiltrating the infected airways. Contrarily, the presence of T cells (CD4^+^ and CD8^+^) and plasmacytoid dendritic cells (DCs, HLA-DR^+^, CD123^+^/CD11c^−^) significantly decrease in peripheral blood of infected children, as compared to healthy children control groups (54, 55). Besides the characterization of the cells infiltrating the airways, the cytokines/chemokine profile observed in the infected tissue is also informative. The main cytokines evaluated in the BALFs of hRSV infected individuals are mainly IL-2, IL-12, IFN-γ, IL-8, IL-6, and TNF-α (35). Importantly, all these cytokines can be evaluated by flow cytometry, ELISA, RT-PCR, or Luminex (56–58).

The types of samples used to detect hRSV or immune-related markers can be nasal washes, nasopharyngeal aspirates (NPA), nasopharyngeal swabs, BALFs, serum and peripheral blood (11, 45, 46). However, cytokines as biomarkers should be assessed at the site of infection (upper and lower respiratory tract) and to a lower extent in peripheric blood. The role of the above-mentioned cytokines during infection is discussed below in the following sections.

## Severity Prognosis in HRSV Infection

Among the patients diagnosed with LRTI, a significant number of hRSV-infected children treated as outpatients will require additional medical attention due to respiratory complications. Furthermore, a significant percentage of diagnosed patients will display recurring wheezing episodes and other complications in the following months after the first LRTI episode (59, 60). It is worth mentioning that these patients can not be identified early after infection due to a lack of accurate tools for predicting disease severity. Furthermore, at present there is no consensus on predicting the outcome of patients with LRTI caused by hRSV, which represents a problem for disease management due to the rapid evolution of the disease in which mechanical ventilation might be unexpectedly required in the course of 24 hours or less (61). Currently, methods that are based on clinical parameters used by physicians are widely accepted to support clinical decisions (62). However, these parameters may be somewhat subjective and are not accurate enough to perform a precise categorization or prognosis of disease severity (63, 64). To address this problem, biomarkers within samples of patients might contribute to a better diagnosis and could help physicians take more accurate decisions, increasing the possibility of obtaining better outcomes (4, 65). In line with this notion, in the last years several research groups have focused on identifying an accurate method for determining the severity and progression of LRTI by hRSV (62, 66–68). Below, we describe diverse parameters and soluble molecules currently used to assess disease severity in hRSV-infected patients (Table 1).

**Table 1:** Molecules and cells as severity markers in respiratory diseases.

**Marker**	**Sample type**	**Market for**	**References**
Viral loads	Nasal washes	Disease progression in hRSV-LRTI.	(69)
Transaminases, aminotransferases and antidiuretic hormones	Serum and nasopharyngeal	Bronchiolitis caused by hRSV	(70, 71)
Lactate dehydrogenase	Nasopharyngeal	Bronchiolitis caused by hRSV in children.	(72, 73)
MUC5AC	Mucus	Severity disease caused by hRSV infection	(74, 75)
Neutotrophins (BDNF and NGF)	BALF	Severity disease caused by hRSV infection	(76)
		Developed asthma later hRSV infection	

*BDNF, Brain-derived neurotrophic factor; NGF, nerve growth factor*.

### Clinical Score as a Biomarker Related to Disease Severity

The use of prediction models to calculate the risk of severe outcome in LRTI in children has been previously implemented based on the clinical characteristics of patients, radiological findings, and laboratory results (77). In the last 10 years, remarkable progress in diagnostics has been achieved thanks to the availability of transcriptional profiles that have allowed establishing fingerprints related to disease progression and severity caused by hRSV infection (78). Among the available methods based on transcriptomic approaches, the “molecular distance to health” (MDTH) has shown to be a promising diagnostic tool for respiratory tract infections (68, 79). The MDTH is a tool designed to measure alterations in the transcriptional profile of immune cells (i.e., neutrophils, cytotoxic cells, and T-cells) obtained from patients (80). Data is obtained from the test as a single score that is compared with a basal score from healthy controls. Importantly, MDTH scores performed during the first days of hRSV infection have been able to predict disease severity in terms of hospitalization days and intensive care requirements (78).

### Microbial Factors as Severity Biomarkers

It is well known that the higher microbial load at the site of infection, the greater the possibility to cause tissue damage, which is related to worse prognosis. Based on this premise, several research groups have tried to demonstrate a direct relationship between viral loads and the severity of the disease (81), but the conclusions are somewhat controversial. Different studies have shown a direct correlation between the increase of viral loads with more severe clinical manifestations (81–83). In fact, these studies showed that high hRSV viral loads at day 3 are significantly associated with requirement for intensive care and respiratory failure (84). In contrast, studies, such as (69, 85) and Piedra et al. have reported the opposite, where high hRSV loads at the beginning of the infection correlate with protective immune response and less severe disease progression (86). These findings raise the discussion about the role of viral loads in disease progression and the possibility of considering this factor as a potential biomarker to determine disease severity in hRSV-LRTI, as viral loads could be leading the host immune response to the virus.

### Soluble Proteins as Biomarkers for Disease Severity

In the last few years, the analysis of protein expression patterns has become one of the most explored fields in diagnosis. The samples used to obtain the protein expression patterns range from blood to nasopharyngeal samples, with both suggesting helpful insights into the identification of molecules related to the severity of the infection. For example, increased levels of serum transaminases, aminotransferases and antidiuretic hormones have been related to severe cases of hRSV bronchiolitis (70, 71). Furthermore, increased levels of lactate dehydrogenase (LDH) in nasopharyngeal samples has also shown to have a predictive value of 88% in determining the severity of the disease in young children with bronchiolitis (72, 73). Another molecule proposed as a disease severity biomarker is mucin 5AC (MUC5AC), a highly glycosylated protein present in the airway mucus (74). This protein has been reported to be detected in nasal aspirates obtained from hRSV-infected children and its presence and concentration is correlated to disease severity caused by hRSV (75). Taken together, several soluble molecules show a correlation with the severity of hRSV-related disease and can be easily detected in samples that are simple to obtain, and thus may be used as biomarkers of disease severity related to infections caused by hRSV.

### Pro-inflammatory Cytokines as Biomarkers for Disease Severity in hRSV Infections

During hRSV infection, the host innate immune response generated against the virus can be unbalanced and ultimately detrimental to the host. Non-optimal responses against the virus are Th2-like responses with the generation of cytokines, which in turn can recruit numerous pro-inflammatory immune cells (35, 56). Furthermore, several studies have reported an increase in the levels of Th2-like cytokines in different types of samples (BALF, serum, blood, plasma, nasopharyngeal, or aspirate washes), which can be correlated with disease severity in children. Such cytokines, which could be used as prognosis biomarkers are IL-33, IL-8, TSLP, IL-6, periostin, and IFN-α. Those biomarkers could predict hRSV disease severity in children (Table 2). Other cytokines, such as IL-12, IL-3, and IL-13 could also be potential biomarkers, although more clinical studies are required (Table 3). Next, we will explain further how some of these cytokines could be useful to predict the severity of hRSV infection.

**Table 2:** Pro-inflammatory cytokines as prognosis biomarkers in respiratory diseases.

**Cytokine**	**Sample type**	**Biomarker for**	**References**
IL-33	Nasal aspirates	Risk for asthma or severe hRSV disease in children after reinfection.	(87)
		Ventilation requirement in infants hospitalized by bronchiolitis caused by hRSV	
	NPA	Bronchiolitis, asthma, and allergic diseases.	(88)
		Allergic inflammation.	(66)
IL-8	Plasma	Predictors of mechanical ventilator requirement during hRSV infection and bronchiolitis.	(4, 48)
	Nasopharyngeal wash	Severity during hRSV infection.	(89)
	Plasma and nasal secretion	Prognosis for children evolving to bronchiolitis by hRSV.	(90)
	Plasma	Severity of airway diseases, asthma and COPD.	(91)
	NPA	Predictive value for the number of days with need of supplemental oxygen.	(92)
TSLP	NPA	Severe bronchiolitis by hRSV.	(93)
	NPA	Increased infant hospitalization and disease severity.	(88)
	BALF	Asthma development by hRSV.	(94, 95)
Periostin	NPA	Severe bronchiolitis by hRSV.	(93)
	NPA	Increased infant hospitalization	(88)
	Bronchial and nasal cells	Persistent or uncontrolled asthma in children.	(96)
	Serum	Persistent or uncontrolled asthma in children.	(97, 98)
	Tracheal aspirates and nasal wash	Pulmonary hypertension and prognosis during hRSV bronchiolitis.	(99)
IL-6	Blood, plasma and serum	Increased infant hospitalization and severe hRSV bronchiolitis.	(4)
	Nasopharyngeal wash	Severity during hRSV infection.	(89)
	NPA	High hRSV disease severity.	(100)
	NPA	Predictive value for the number of days with need of supplemental oxygen.	(35, 92)
IFN-α	Blood	Severity of the disease in children under 2 years infected by hRSV.	(101)
	Blood and nasopharyngeal swabs	More severe illness and recurrent wheezing in in hRSV bronchiolitis.	(89, 102)

*BALF, Bronchoalveolar lavage fluid; NPA, Nasopharyngeal aspirate*.

**Table 3:** Pro-inflammatory cytokines as potential prognosis biomarkers in respiratory diseases.

**Cytokine**	**Sample type**	**Potential Biomarker for**	**References**
IL-12	BALF	Recurrent wheezing due to hRSV infection.	(66)
		Developed asthma later hRSV infection.	
		Developed asthma in infants with bronchiolitis caused hRSV infection.	(35)
IL-3	BALF	Recurrent wheezing due to hRSV infection.	(66)
		Developed asthma later hRSV infection.	
	NPA	Severe bronchiolitis by hRSV.	(100)
IL-13	Nasal aspirates	Ventilation requirement in infants hospitalized by bronchiolitis caused by hRSV.	(87)
	Blood	Asthma diagnosis.	(100)
	Nasal washes	High IL-13 levels are elevated in children with hRSV LRTI.	(35)

*BALF, Bronchoalveolar lavage fluid; NPA, Nasopharyngeal aspirate*.

#### Interleukin-33 (IL-33)

IL-33 is constitutively expressed by endothelial and epithelial cells. The main function of this cytokine is the initiation and development of the innate and adaptive Th2 type immune response (103). Cells expressing the ST2 receptor respond to IL-33, including mast cells, eosinophils, and basophils, among others (104). Type-2 innate lymphoid cells (ILC2s) are also targeted by IL-33 to produce Th2-type cytokines (IL-6, IL-8, IL-5, IL-13), which in turn promote a Th2 response with eosinophil recruitment, generating an exacerbated disease (105) (Figure 1B). Recent studies with mice in which IL-33 was neutralized during hRSV infection, showed that severe pathology was not induced and that mice treated with IL-33 during hRSV infection quickly developed the disease, resulting in more severe clinical outcome (35, 88). Interestingly, Saravia et al. measured IL-33 levels in NPA and showed a link with ventilation requirement in infants hospitalized by bronchiolitis caused by hRSV (87). In 2015, Bertrand et al. performed a study in children with bronchiolitis caused by hRSV and detected high levels of IL-33 expression levels in NPA in patients with a family history of atopy (66). García-García et al. measured IL-33 levels from NPA in children infected with hRSV, associating bronchiolitis with high levels of this cytokine. Furthermore, both studies describe that IL-33 cytokine is elevated when coinfection occurred (88). Taken together, these results indicate that IL-33 could be a good biomarker to determine the severity and prognosis during bronchiolitis caused by hRSV.

#### Interleukin-8 (IL-8)

IL-8 has a mayor chemotactic role, and is mainly produced by monocytes, endothelial cells, macrophages, and T cells (106, 107). IL-8 binds to G protein-coupled receptors CXCR1 and CXCR2 expressed by cells that include monocytes, neutrophils, endothelial cells, macrophages, and T cells, among others (108, 109) (Figure 1B). During an infection with hRSV, McNamara et al. found that the concentration of IL-8 remains elevated during the disease, even when the number and recruitment of neutrophils ultimately decreased (110). Elevated IL-8 levels (in nasopharyngeal samples) have been widely correlated with disease severity caused by hRSV infection, including the risk of mechanical ventilation (4, 90). In 2013, Díaz et al. found high IL-8 levels in NPA in children with severe hRSV bronchiolitis as compared to controls and patients with mild disease manifestations. More specifically, they observed an increase in IL-8 in a group of patients with severe disease (111), which may suggest that higher levels of this cytokine relate to higher severity of hRSV infection. Tabarani et al. identified in nasopharyngeal washes increased levels of IL-6, IL-8, and TNF-α associated to hRSV disease severity in young children (89). In this study, the authors associated the severity of disease with the age of the individuals, chronic diseases and elevated concentrations of IL-8, as well as other molecules (89). In another study, which was performed in children with severe hRSV infection, Brand et al. assessed the levels of IL-8 in plasma and NPA and found an increase in IL-8 in the plasma of children with severe disease, as compared to children with mild or moderate disease (48). In 2015, Díaz et al. performed another study in children with bronchiolitis caused by hRSV and Rhinovirus (RV). This study showed higher IL-8 levels in NPA of children infected with both, hRSV and RV than children infected with RV alone, which was associated with more days requiring O_2_ treatment (92). Based on this study, it can be suggested that high IL-8 levels in children infected hRSV will act as a good predictor for determining the days that requiring O_2_ treatment. In 2016, Huang et al. performed a clinical study that included 96 patients with asthma-chronic obstructive pulmonary disease (COPD) and 35 healthy controls. Their results showed an increment of IL-8 and other cytokine levels that were related to the severity of airway diseases. The researchers suggest that IL-8 could be a potential marker for the evaluation of asthma and COPD (91). There are not new clinical studies that correlate high levels of this cytokine with the disease severity.

#### Thymic Stromal Lymphopoietin (TSLP)

TSLP is expressed by several cell types, but mainly by epithelial cells and keratinocytes (112, 113). Two isoforms have been described for this cytokine: a long and a short form of TSLP (114). The short isoform is constitutively expressed in several tissues, particularly in those that are highly sensitive to inflammation. Importantly, the long isoform of TSLP has been widely correlated with exacerbated immune responses and the establishment of allergic and asthma in patients with atopic dermatitis (95) (Figure 1B). Asthma may result as a consequence of different factors in children. However, a possible association with viral infections has gained increased attention of researchers in the last decade (88). At present, there is increasing evidence suggesting an association between TSLP elicited upon infection with hRSV or RV and the development of asthma (88, 94). However, it still remains to be elucidated whether asthma favors severe viral disease or if asthma is the result of severe disease elicited during respiratory infection. Lee et al. reported that viral antigen recognition triggers a signaling cascade involving the NF-κB nuclear factor and retinoic acid induced gene 1 (RIG-1) (115). The activation of this cascade resulted in TSLP production and a strong Th2 response, contributing to the pathophysiology observed in severe bronchiolitis, which eventually in some cases progressed to asthma (115). Later, García-García et al. showed an association between TSLP, together with periostin and IL-33, with disease severity in the infection of the respiratory tract of children. This study showed a correlation between increased levels of TSLP with hRSV bronchiolitis and coinfections with rhinovirus, as well as with severe disease and intensive care unit (ICU) admission (88).

#### Interleukin-6 (IL-6)

IL-6 is a soluble mediator that can be produced by macrophages and epithelial cells (116). After its synthesis, IL-6 moves to the liver through the bloodstream and generates a pleiotropic effect over immunity and inflammation (117). This cytokine is involved in the promotion of the differentiation of naïve CD4^+^ and CD8^+^ T cells and is an important link between innate and acquired immunity (117) (Figure 1B). In 2013, Tabarani et al. evaluated the levels of IL-6 in nasopharyngeal wash samples from children with LRTI and hRSV. Interestingly, they found a correlation between the magnitude of the clinical manifestations elicited by hRSV infection and high levels of IL-6 amongst other inflammatory mediators (CCL2, TNF-α, CXCL8, IL-10) (89). On the other hand, Brown et al. have suggested that high levels of IL-6 in the plasma could indicate a higher probability of infant hospitalization and severe bronchiolitis caused by hRSV (4). In 2016, Lu et al. also detected high levels of IL-6 in NPA of patients with hRSV and this was correlated with higher hRSV disease severity (100). Increased levels of IL-6 and other cytokines have also been found in nasal lavage fluids of children with LRTI, particularly those which needed O_2_ treatment (35, 118). Other studies performed in children with bronchiolitis caused by hRSV infection showed that high IL-6 levels in nasal samples and BALF correlated with the need for ventilation and with a higher degree of hypoxia (35, 92). In this study, the authors suggested that IL-6 and other cytokines assessed could be reliable biomarkers to determine the severity of hRSV infection.

#### Periostin

Periostin is a protein that is expressed at basal levels in almost all human tissues (119). Its expression is also found in the respiratory epithelium and is elevated levels in asthmatic children (120). This protein is produced by eosinophils in response to IL-4 an IL-13 signaling (121) (Figure 1B). The role of periostin is related to the generation of allergic inflammation and the development of a Th2 phenotype, among others (120) (Figure 1B). Periostin has been associated with asthma severity and increased levels of periostin have been found in the serum of children with exacerbated manifestations of asthma (122). Lopez-Guisa and colleagues evaluated periostin levels in bronchial and nasal cells from asthmatic, non-asthmatic, atopic, and healthy children and found a significant increase in periostin levels in asthmatic children (3.7 times), as compared to the other groups (96). These results were confirmed in studies that showed a correlation between high levels of periostin in the serum with persistent or uncontrolled asthma in children (97, 98). In fact, clinical manifestations of asthma are considered to be very similar to bronchiolitis symptoms (123). These findings suggest that asthma could be a sequel of severe bronchiolitis in children (123). García-García et al. showed in NPAs that increased concentrations of periostin were associated with more severe hRSV infection, as compared to healthy children (88, 93). More recently, periostin levels were associated with the severity of viral bronchiolitis, as children with severe pulmonary hypertension had high levels of this protein as compared to children with mild pulmonary hypertension (8,887 ± 1,582 pg/ml vs. 5,016 ± 1,017 pg/ml) (99). These results indicate that periostin could be another good biomarker for the prognosis of hRSV infection and particularly bronchiolitis.

#### Interferon Alpha (IFN-α)

IFNs are a large family of pleiotropic cytokines. Particularly, IFN-α and IFN-β are type-I interferon family members produced by epithelial cells and most of immune cells (124). To exert its biological action, type-I IFNs binds to the type-I IFN receptor (IFNAR1/2) (125), which triggers the expression of pro-inflammatory molecules and antiviral genes, such as those involved in the degradation of viral RNA (126). Importantly, the recognition of the hRSV non-structural protein 1 (NS1) has been correlated with impaired IFN-α function, particularly through the induction of the miRNA miR-29a, which inhibits the expression of the IFN-α receptor in infected cells (101). These studies suggest that low levels of IFN-α could be related to the severity of hRSV infection and hence could be used as a biomarker. However, other studies based on the transcriptional profile of blood samples and nasopharyngeal swabs, report contrasting results, indicating that type-I interferons, particularly IFN-α/β are increased in hRSV bronchiolitis and correlate with severe illness and recurrent wheezing (89, 102). These studies suggest that interferon signaling pathways may serve as important biomarkers associated to hRSV loads and severity (102). Resolving the discrepancies found among different studies analyzing the role of IFN-α in hRSV disease severity will require further investigations that ideally relate transcriptional findings with protein levels in blood and nasopharyngeal samples.

## Other Potencial Pro-Inflammatory Cytokines as Biomarkers for Severity Caused by HRSV

Besides the cytokines described above as potential biomarkers for hRSV severity (4), recent studies have preliminarily pointed out other pro-inflammatory cytokines that show positive correlations with hRSV severity and are potential prognosis biomarkers for respiratory diseases (Table 3). Some of these cytokines are described below.

### Interleukin-12 (IL-12)

IL-12 is produced in response to viral or bacterial infections by DCs and other antigen-presenting cells and is involved in promoting naïve T cell differentiation into Th1 T cells (127) (Figure 1B). Bertrand et al. have shown that nasal and lung samples display increased levels of IL-12 in LRTI patients. Furthermore, they showed for first time that high levels of IL-12p40 (in BALF) and other cytokine could be correlated with recurrent wheezing and the development of asthma in infants with bronchiolitis caused by hRSV infection (35, 66).

### Interleukin-3 (IL-3)

IL-3 is mainly expressed by mast cells and activated T cells located in the airways (128). This cytokine induces an increase in basophil and eosinophil production (129) (Figure 1B) and is involved in the pathogenesis of asthma (128). In 2015, Bertrand et al. described for the first-time the presence of high levels of IL-3 in BALF and NPA obtained from children < 9 months with acute bronchiolitis caused by hRSV. Furthermore, the authors found a correlation between high levels of IL-3 with episodes of recurring wheezing and the development of asthma in the future (66). Lu et al. also found high levels of IL-3 in NPA in children with bronchiolitis caused by hRSV and an increased risk of asthma, which was associated with higher disease severity (100). The results of this study suggest that IL-3 could be involved in the development of chronic airway inflammatory diseases and that it could be used to predict clinical outcomes in hRSV-LRTI. Consistently, the authors suggested that IL-3 could be eventually used to predict the clinical outcome of patients.

### Interleukin-13 (IL-13)

In the lungs, IL-13 is the mediator of eosinophilic inflammation, mucosal secretion, and bronchial hyper reactivity (130). It has been observed that IL-13 is elevated in COPD, as well as in asthma and other lung diseases (131). Importantly, IL-13 is produced in response to IL-33 signaling and is released from various cells, including alveolar macrophages, basophils, mast cells, eosinophils, ILC2 and CD4^+^ T cells (132) (Figure 1B). In 2015, Saravia et al. linked high levels of IL-13 and IL-33 with the requirement for ventilation in infants hospitalized with bronchiolitis caused by hRSV (87). Consistently, in an animal model of hRSV (BALB/c mice), an up-regulation of IL-13 has been reported, which results in the recruitment of eosinophils to the airways that generates exacerbated mucus production, lung hyperreactivity and airway inflammation (132). A more recent study performed in 2016 evaluated IL-13 levels in the blood of children being treated for respiratory symptoms following severe hRSV bronchiolitis and found that IL-13 could be used as a clinical asthma diagnosis marker (100).

## Concluding Remarks

Biomarkers for classifying the severity of respiratory tract infections have become a global need due to the lack of effective strategies to decrease the impact of such diseases and the need for improving the management of patients and their potential outcomes. Most efforts point to the development of highly sensitive, rapid, and low-cost techniques that allow predicting in an accurate way the prognosis of patients with respiratory infections. Nowadays, an important number of molecules have been identified which could help asses disease severity, however their specificity and sensitivity remain challenging and are not strong enough yet to accurately predict disease outcome and become a canonic biomarker for predicting LRTI severity associated to hRSV. Hence, more studies are needed to establish the pro-inflammatory cytokine and cytokine expression patterns that are related to disease development during the different stages of hRSV infection. Ideally, particular pro-inflammatory cytokine profiles will ultimately allow determining early on during infection the severity of disease caused by hRSV.

## Author Contributions

YV, LG, and LN are responsible for the writing of this review article. PG, CR, and PB are responsible for reviewing the article and SB is the leading investigator and assisted in the organization and revision of the manuscript.

### Conflict of Interest Statement

The authors declare that the research was conducted in the absence of any commercial or financial relationships that could be construed as a potential conflict of interest.

## Abbreviations

hRSV: Human respiratory syncytial virus
LRTI: Lower Respiratory Tract Infection
TSLP: Thymic Stromal Lymphopoietin
IL: Interleukin
IFN: Interferon
BALF: Bronchoalveolar lavage fluid
NPA: Nasopharyngeal aspirate
TLR: Toll-like receptors
AECs: Airway epithelial cells
NF-κB: Nuclear factor κB.
